# Use of Inulin and Pumpkin Oil in the Manufacture of High-Quality Mortadella-Style Sausage from Buffalo Meat

**DOI:** 10.3390/foods14081427

**Published:** 2025-04-21

**Authors:** Silvia Jane Lombardi, Filomena Nazzaro, Luigi Grazia, Raffaele Coppola, Florinda Fratianni, Michela Pellegrini, Ilenia Iarusso, Patrizio Tremonte, Francesca Coppola

**Affiliations:** 1 Department of Agricultural Environmental and Food Sciences (DiAAA), University of Molise, Via de 8 Sanctis snc, 86100 Campobasso, Italy; 2 Institute of Food Science Italian National Research Council, Via Roma 64, 83100 Avellino, Italy; 3 Department of Agricultural and Food Sciences, Alma Mater Studiorum, University of Bologna, p.zza Goidanich 60, 47521 Cesena, Italy; 4 Department of Agricultural, Food, Environmental and Animal Science, University of Udine, Via Sondrio 2A, 33100 Udine, Italy; 5 Department of Agricultural Sciences, University of Naples “Federico II”, Piazza Carlo di Borbone 1, 80055 Portici, Italy

**Keywords:** bio-preservation, fat reduced, plant-based ingredients, sensory profile

## Abstract

The growing demand for healthier meat products has driven the reformulation of processed meats to reduce saturated fat while preserving sensory and technological attributes. Buffalo meat (*Bubalus bubalis*), with its high protein content, low intramuscular fat, and favorable fatty acid profile, offers a promising base for healthier formulations. However, its fat content may compromise texture, juiciness, and flavor, necessitating strategies to optimize product quality. This study investigated the effects of replacing pork fat with inulin and pumpkin seed oil in a cooked buffalo meat product, focusing on compositional, oxidative, microbiological, and sensory parameters. Two plant-based ingredients were selected: inulin from chicory, used as a fat mimic due to its gel-forming ability, and pumpkin seed oil, a structural analog with antimicrobial activity. Preliminary trials identified optimal concentrations for balancing technological and functional performance. A 2% inclusion of pumpkin seed oil, exceeding its in vitro MIC (0.4–1.5%), ensured effectiveness in the food matrix. Reformulated products exhibited significantly reduced fat (*p* < 0.05), enhanced fiber, and a lipid profile rich in polyunsaturated fatty acids (>45%), qualifying for European Union health claims. Oxidative stability improved (*p* < 0.01), and sensory analysis revealed enhanced aroma complexity, with nutty and roasted notes. Microbiological assessments confirmed a protective effect against spoilage bacteria. These results support the development of a nutritionally improved, microbiologically safer cooked product, such as mortadella-style sausage, while also offering strategies for broader innovation in reformulating functional meat products.

## 1. Introduction

The food industry is focusing on the careful selection of high-quality raw materials, beneficial microorganisms, and state-of-the-art processing methods to enhance food safety, overall quality, and environmental sustainability [1,2,3]. Consumer preferences have shifted considerably, with rising demand for food products that should be natural and subject to minimal processing [4], rich in nutrients and functional components [5], produced through environmentally sustainable practices [6,7], and free from, or containing only minimal amounts of additives and preservatives [8]. This trend has also influenced the processed meat sector, which offers a wide range of culinary specialties. Meat products present an excellent opportunity for the incorporation of bioactive compounds to create functional foods and help mitigate the adverse effects of excessive consumption [9,10]. A key strategy for these products is reducing saturated fat content while preserving their distinct sensory and technological properties, as well as enhancing their health benefits [11,12,13]. For this purpose, various approaches can be used. One possible strategy is the partial or complete replacement of pork fat with functional ingredients, including vegetable oils and fibers, which could introduce new and beneficial nutritional and health properties to the product [14,15,16]. Buffalo meat (*Bubalus bubalis*) stands out as a promising option for reformulated meat products for its specific features [17,18,19,20]. Nevertheless, due to its reduced fat content, buffalo meat might require modifications in processing to maintain its moisture, texture, and taste attributes typically associated with meats with a higher fat content [21]. One approach to offset fat reduction is adding inulin, a naturally occurring fructan known for its prebiotic benefits [22]. Several studies showed that inulin can improve the water retention capacity, emulsion stability, and sensory acceptability of reduced-fat meat products [23,24,25]. Inulin has been successfully incorporated into products made from pork, beef, chicken, and turkey, improving nutritional quality without compromising sensory acceptability [15,26,27]. The combined use of buffalo meat as a lean substitute and inulin as a fat replacer in processed meat products has been explored only to a limited extent. Furthermore, the incorporation of vegetable oils into meat products represents a multifaceted approach thanks to their antioxidant and antimicrobial properties [28]. Again, proposals for using oils or other natural substrates with antioxidants, antimicrobial properties, or prebiotic activity [29,30,31,32,33] are attracting considerable interest in the food field. This approach becomes even more effective when vegetable oils are combined with other natural substances with similar or complementary properties, creating a synergistic effect that can further improve product performance [34,35]. An appropriate selection process not only enhances the sensory qualities of the meat products but also significantly improves their safety and shelf life [36,37,38]. Pumpkin seed oil has been scientifically validated to exhibit a range of beneficial properties, including antioxidants, anti-inflammatory, and antimicrobial effects [39,40,41]. According to Fratianni et al. [42], its antioxidant properties could mitigate oxidative stress in food products, potentially extending their shelf life and enhancing nutritional value. One of the most celebrated meat products in the culinary landscape, both in Italy and internationally, is mortadella, a highly favored product that is renowned for its distinct sensory qualities and culinary adaptability. Mortadella, or cooked sausage mortadella style, has a very low carbohydrate content, making it suitable for low-carb diets. It is a source of B vitamins, particularly B1 (thiamine), B2 (riboflavin), and B12 (cyanocobalamin), as well as minerals like iron and zinc [43,44]. For the above reasons, among cooked meat, mortadella can be considered an excellent candidate as a base for developing a new food product, with improved functional properties while still preserving all its characteristic features of typicity. In previous work, we evaluated the use of pumpkin seed oil to produce Napoli-type salami made from buffalo meat [45]. This study evaluated the effects on the texture, color, lipid oxidation, sensory, and microbiological properties of partially replacing pork fat with inulin and pumpkin seed oil in the formulation of a buffalo meat sausage.

## 2. Materials and Methods

### 2.1. Fat Replacer

Two plant-based ingredients were selected as fat substitutes: a structural analog (vegetable oil) and a functional mimic (fiber). Specifically, in liquid form, a light yellow pumpkin seed oil (Merck Life Science S.r.l., Milan, Italy) was also chosen as a plant analog because of its antimicrobial activity [42]. Inulin obtained from chicory (purity >90%) (Merck Life Science S.r.l., Milan, Italy) was utilized as a mimic fat replacer due to its ability to form a gel-like matrix upon hydration. Inulin, just before use, was suspended in water (1:1 *w*/*v*), shaken at 80 °C for 5 min, and cooled to a gel-like consistency at 4 °C. Pumpkin seed oil and inulin concentrations were optimized through preliminary trials to balance technological, nutritional, and bioactive functionality.

### 2.2. Antimicrobial Effect of Pumpkin Seed Oil

Pumpkin seed oil, previously characterized for its biological properties [42,45], was evaluated for its antimicrobial activity against *Pseudomonas putida* DSMZ 291T, *Pseudomonas fluorescens* DSMZ 50009T, *Pseudomonas fragi* DSMZ 3456T, *Brochothrix thermosphacta* DSM 20171T, and *Clostridium sporogenes* DSM 795T, obtained from the DSMZ–German Collection of Microorganisms and Cell Cultures (Braunschweig, Germany). *Pseudomonas* spp. and *Brochothrix thermosphacta* were cultivated at 28 °C in Nutrient Broth (Oxoid, Milan, Italy), and *C. sporogenes* was cultivated at 28 °C in Reinforced Clostridium Medium (Oxoid, Milan, Italy). The antimicrobial activity of the extract was tested using the agar well diffusion assay, following the method described by Tremonte et al. [46,47]. The minimum inhibitory concentration (MIC) was determined using the agar dilution method, as outlined in EUCAST document 3.1 [48] and adapted by Lombardi et al. [10].

### 2.3. Cooked Sausage Preparation

The lean meat of male Mediterranean buffalo (*Bubalus bubalis*), which were about 400–450 days old, was utilized for Mortadella-style cooked sausage preparation. The selected cuts, including chuck and brisket, underwent meticulous trimming to eliminate tendons and connective tissue before being diced into 10 cm cubes and cooled to 0–2 °C before processing. Sausage formulations were standardized to contain buffalo meat (50%), pork belly (30%), and ice (10%), producing four experimental batches (9 kg each). Specifically, 5 kg of buffalo meat and 3 kg of pork belly were processed using a cutter, operating at a controlled speed for 10 min to prevent the mixture temperature from exceeding 10 °C. During the first minute of mincing, the following ingredients were incorporated: sodium chloride (1.5%), sodium nitrite (0.008%), ascorbic acid (0.04%), dextrose (0.8%), and a standardized spice blend (1%), composed of black pepper, garlic, coriander, and *Myristica fragrans* arillus. The formulations were differentiated into four experimental groups based on the fat replacement strategy:

- C (Control): Included cubed pork lard (10%) (1 cm edge), pretreated by immersion in boiling water (90 °C, 5 min) to remove low-melting-point fats, followed by drying with a sterile cotton cloth.
- P: Incorporated pork lard (8%) and pumpkin seed oil (2% *v*/*w*) as a partial fat replacer.
- I: Contained pork lard (4%) and inulin (6% *w*/*w*) as a partial fat replacer.
- IP: Included pork lard (2%), inulin (6% *w*/*w*), and pumpkin seed oil (2% *v*/*w*).

Each batch was stuffed into casings using a pneumatic filler and subjected to steam cooking until reaching a core temperature of 71 °C. A subsequent browning phase was conducted at 80 °C for 7 min, followed by rapid cooling in a blast chiller and vacuum packaging for storage. Samples from each batch of the mortadella-style sausages were stored at a refrigerated temperature (+5 °C) for a period of 120 days.

### 2.4. Sampling

Samples were taken from each batch before and after cooking at different storage times (0, 30, 60, 90, and 120 days). Specifically, the samples were taken as detailed below. During the stuffing process (between the stuffing of the first and second samples, between the second and third batches, and between the third and fourth samples of each batch) an aliquot (300 g) of mixed meat representative of each batch was collected and immediately placed in sterile polyethylene bags, transported in refrigerated conditions (4 °C) to the laboratories, and subjected to subsequent analysis: one aliquot was immediately subjected to microbiological analysis, while the other was frozen at −20 °C for subsequent chemical analysis. As regards the samples after cooking, the four cooked cold cuts from each batch were portioned into 500 g aliquots and vacuum-packed, resulting in 20 pieces from each batch. Two samples were collected from each batch at the time of packaging and at regular 30-day intervals during the four-month storage period. The samples were subjected to microbiological, chemical, and sensory analysis.

### 2.5. Chemical, Physicochemical, Microbiological, and Sensorial Analyses

#### 2.5.1. Chemical Characterization

The following determinations were conducted according to AOAC [49]: moisture (official method 958.14), protein (official method 928.08), lipids (official method 960.39), ash (official method 920.153), and fiber (official method 985.29). The carbohydrate content (%) was estimated by difference, applying the following equation:

C% = 100 − (M + P + L + A + F)

where M represents the moisture content (%); P denotes the protein content (%); L corresponds to the lipid content (%); A signifies the ash content (%); F indicates the fiber content (%).

In addition, the fatty acid profile was determined by high-resolution gas chromatography (HRGC) using a GC Trace 1600 gas chromatograph (ThermoQuest EC Instruments, Milan, Italy). Fatty acid methyl esters (FAMEs) were prepared via the transesterification of approximately 25 mg of previously extracted lipids. The lipids were first dissolved in 2 mL of petroleum ether and subsequently reacted with 2 mL of boron trifluoride-methanol (BF₃-methanol) reagent to facilitate esterification. Individual fatty acids were separated and quantified using a capillary gas chromatographic system equipped with a flame ionization detector (FID) and a highly polar column specifically designed for fatty acid analysis. The concentrations of saturated fatty acids (SFAs), monounsaturated fatty acids (MUFAs), and polyunsaturated fatty acids (PUFAs) were determined based on the relative peak areas of the corresponding FAMEs, following the methodology described by Tremonte et al. [50]. The identification of individual fatty acids was achieved by comparing retention times with those of authenticated standard mixtures of fatty acid methyl esters (FAME Supelco, Sigma-Aldrich, Milan, Italy). All measurements were conducted in triplicate to ensure analytical accuracy, and results were expressed as a percentage of total fatty acids.

#### 2.5.2. Lipid Peroxidation

The assessment of oxidative stability in cooked salami was conducted through the quantification of thiobarbituric acid reactive substances (TBARSs). The analysis followed a modified thiobarbituric acid (TBA) assay [50]. A homogenized sample (0.5 g) was mixed with 2.5 mL of a reagent solution containing 0.375% TBA (*w*/*v*), 15% trichloroacetic acid (TCA) (*w*/*v*), and 0.25 M hydrochloric acid (HCl). The mixture was subjected to thermal treatment in a boiling water bath (95–100 °C) for 10 min to promote chromophore formation, then rapidly cooled under running water. After centrifugation at 3600× *g* for 20 min at 25 °C, the absorbance of the supernatant was measured at 532 nm using a spectrophotometer (BioSpectrometer Basic, Eppendorf, Hamburg, Germany). A standard calibration curve was generated using 1,1,3,3-tetraethoxypropane (TEP) at concentrations ranging from 0 to 6 ppm, ensuring precise quantification. TBARS values were expressed as milligrams of malondialdehyde (MDA) per kilogram of sample (mg MDA/kg), quantitatively measuring lipid oxidation in cooked sausage.

#### 2.5.3. Microbiological Analyses

Microbiological analyses were performed in duplicate on two samples from each batch at 0, 30, 60, 90, and 120 days of storage. In detail, about 10 g of each sample was weighed aseptically and subjected to serial decimal dilution in a sterile salt solution (NaCl 0.9%). Homogenization was performed with a Stomacher 400 (Seward Medical, London, UK) for 3 min to ensure uniform microbial dispersion. Appropriate dilutions were plated on selective and nonselective media for enumeration of the following microbial groups: Enterococci, *Brochotrix thermosphacta*, total coliforms, *Pseudomonas* spp., *Clostridia,* and *Eumycetes*, detected after proper incubation in appropriate media and conditions, as described in previous studies [47,51]. Homogenization was performed with a Stomacher 400 (Seward Medical, London, UK) for 3 min to ensure uniform microbial dispersion. Appropriate dilutions were plated on selective and non-selective media to enumerate the following microbial groups: Enterococci (Slanetz and Bartley agar, 37 °C for 48 h), *Brochothrix thermosphacta* (STAA agar, 25 °C for 48 h), total coliforms (VRBA, 37 °C for 24 h), *Pseudomonas* spp. (*Pseudomonas* Agar Base with CFC supplement, 25 °C for 48 h), *Clostridia* (TSC agar under anaerobic conditions, 37 °C for 24–48 h), and *Eumycetes* (Sabouraud Dextrose Agar, 25 °C for 5 days).

#### 2.5.4. Sensorial Characterization

Sensorial analysis was performed according to the protocol suggested by Chiavari et al. [52]. The sensory assessment of sausages was conducted by a panel of 20 trained evaluators, aged between 22 and 65 years, with specialized expertise in meat sensory analysis, particularly in sausage evaluation. The panelists were selected from an initial pool of 40 candidates following 10 training sessions, ensuring consistency and reliability in their assessments. All participants provided their informed consent (Document S1). Under the supervision of a panel leader, the evaluators performed three independent sensory sessions in a controlled environment. Each judge was anonymously presented with one sample per batch, consisting of a single slice of cooked sausage. To prevent bias, the samples were coded with randomized alphanumeric identifiers that did not reveal any information about the origin batch. A 9-point sensory scale was used to evaluate key attributes, including odor, aroma, flavor, color, and texture (Table S1). The assessment of odor and aroma encompassed both intensity and dominant descriptors. Predefined descriptor categories included meat-related aromas (meat broth, fresh meat, sour meat, fat, aged meat); animal-derived notes 0 (stable, gut, leather); spice-associated aromas (green pepper, garlic, cinnamon, red pepper, nutmeg); and other sensory perceptions (dried fruits, dairy, vanilla, floral, roasted, nutty, licorice, kefir, mold, rancid, ammonia). Additionally, panelists were allowed to identify up to two supplementary aroma descriptors beyond the predefined categories, facilitating a more comprehensive characterization of the product’s aromatic profile. Flavor evaluation focused on the intensity of salty, acidic, and bitter components, while trigeminal sensations were analyzed with particular attention to spiciness. Color assessment considered both intensity and uniformity. Texture analysis encompasses multiple parameters: firmness, elasticity, hardness, moisture content, and chewability. This structured approach to sensory analysis ensured a systematic and reproducible evaluation of the sausages’ organoleptic properties.

### 2.6. Statistical Analyses

Data analysis was performed using GraphPad Prism 10.4.1. The minimum inhibitory concentration of pumpkin seed oil against target microorganisms, the evolution of chemical parameters throughout the ripening period, and the sensory attributes assessed at the end of storage were analyzed using analysis of variance (ANOVA), followed by Bonferroni post hoc tests to determine statistically significant differences (*p* < 0.05 or *p* < 0.01) among the different cooked sausage formulations. Additionally, lipid and fiber content were evaluated using an independent *t*-test, comparing batches produced with and without pumpkin seed oil fortification to assess significant differences (*p* < 0.05 or *p* < 0.01). Finally, frequency analysis of key descriptors used for aromatic perceptions was conducted and visualized using a word cloud generator to illustrate the most recurrent sensory terms.

## 3. Results and Discussion

### 3.1. Antimicrobial Effect of Pumpkin Seed Oil on Selected Bacterial Strains

**Figure 1:**
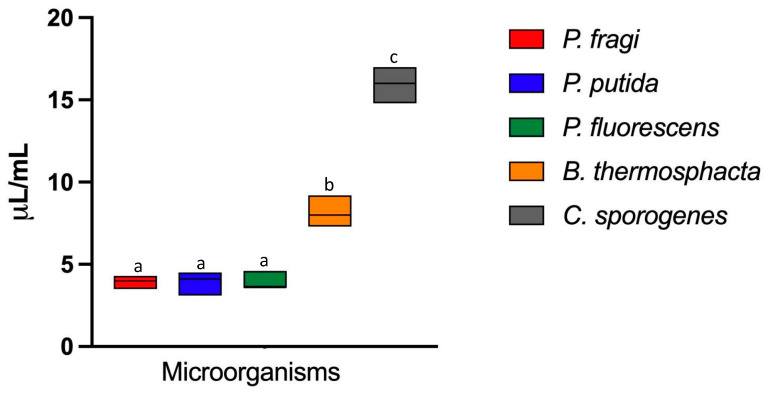
Minimum inhibitory concentration (MIC expressed as μL/mL) of pumpkin seed oil against *Pseudomonas putida* DSMZ 291T, *Pseudomonas fuorescens* DSMZ 50009T, *Pseudomonas fragi* DSMZ 3456T *Brochothrix thermosphacta* DSM 20171T, and *Clostridium sporogenes* DSM 795T. The mic values of five replicates against each microorganism are shown in the box, and the median is shown as a line. Different letters indicate significant differences (*p* < 0.01) detected by ANOVA test.

The antimicrobial activity of pumpkin seed oil was evaluated against three Gram-negative bacterial species, *P. fragi*, *P. fluorescens*, and *P. putida*, and two Gram-positive species, *B. thermosphacta* and *C. sporogenes*. The findings revealed that all tested strains exhibited a certain degree of sensitivity to the oil, although notable differences emerged in terms of susceptibility levels (Figure 1). *Pseudomonas* species were the most susceptible among the tested microorganisms, with a minimum inhibitory concentration (MIC) of 4 µL/mL. This result aligns with previous studies suggesting that *Pseudomonas* species are highly susceptible to plant-derived antimicrobial compounds, likely due to their outer membrane composition, which may facilitate the penetration of bioactive compounds [53,54,55]. Other authors, also studying different extracts, have found increased sensitivity in strains of gram-negative species [56,57]. Conversely, *B. thermosphacta* DSM displayed a moderate sensitivity to pumpkin seed oil, requiring a MIC of 8 µL/mL. This value indicates a significantly (*p* < 0.01) lower susceptibility compared to *Pseudomonas* spp., possibly due to differences in cell wall structure and metabolic pathways that influence resistance to essential oils [58]. Notably, *C. sporogenes* exhibited the highest level of resistance among the tested strains (MIC = 16 µL/mL, *p* < 0.01).

The increased resistance observed in *C. sporogenes* can likely be attributed to its intrinsic resistance and ability to withstand adverse environmental conditions, including exposure to antimicrobial agents [59]. Nevertheless, the inhibition of *C. sporogenes*, despite exhibiting significantly (*p* < 0.01) higher minimum inhibitory concentration (MIC) values compared to *Pseudomonas* spp. and *B. thermosphacta*, represents a noteworthy achievement in the meat industry, particularly for cooked meat products. Previous studies have also reported promising anti-*Clostridium* activity associated with vegetable oils [60]. These findings further support the antibacterial efficacy of pumpkin seed oil against various meat-contaminating bacterial species, including those commonly found in buffalo meat [20,47,61]. Consequently, this reinforces its potential application in biocontrol strategies aimed at improving microbial safety and the preservation of processed meat products.

### 3.2. Pumpkin Seed Oil Amount for Validation in a Cooked Buffalo Meat-Based Sausage

The selection of the optimal concentration of pumpkin seed oil for validation in a complex food system, such as cooked buffalo meat salami, was driven by its dual functional role as a partial substitute for pork fat and as an antimicrobial agent. Pumpkin seed oil was intended not only to improve the lipid profile of the product but also to exert a protective antimicrobial effect, so its inclusion level was carefully determined to exceed the minimum inhibitory concentration (MIC) observed in vitro. In antimicrobial studies, the MIC represents the lowest concentration at which microbial growth is inhibited under controlled laboratory conditions. However, food matrices are highly complex systems in which interactions with proteins, lipids, and other constituents can significantly influence the bioavailability and efficacy of antimicrobial compounds [62]. As a result, bioactive compounds are often incorporated at concentrations above their MIC values to ensure sufficient antimicrobial activity in real food applications [7,10,63,64]. Considering that the MIC values against the targeted microbial strain ranged from 0.4% to 1.5% (i.e., from 4 mL/L to 15 mL/L), a pumpkin seed oil concentration of 2% was selected for validation in the cooked buffalo meat salami. This level was deemed appropriate to compensate for potential interactions within the food matrix and cooking process [65] while ensuring an effective antimicrobial effect.

### 3.3. Effect of Inulin and Pumpkin Seed Oil on Chemical Features

**Figure 2:**
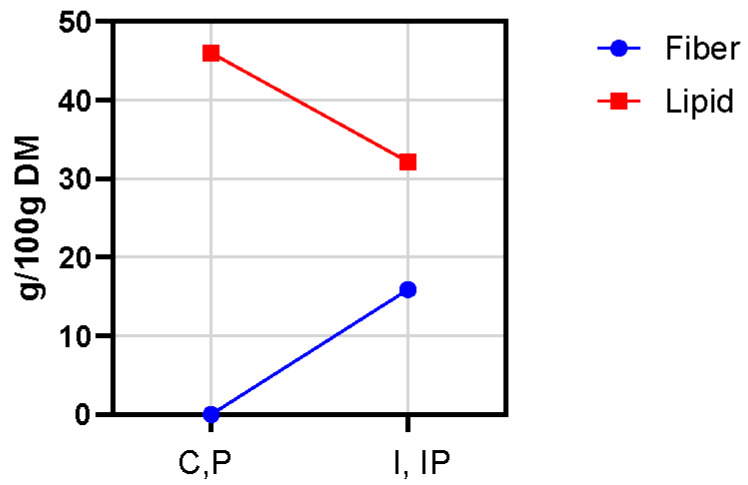
Lipid and fiber content in samples of different cooked sausages produced without inulin (C: 10% pork lard; P: 8% pork lard plus 2% *v*/*w* pumpkin seed oil) or with inulin (I: 4% pork lard plus 6% *w*/*w* inulin; IP: 2% pork lard plus6% *w*/*w* inulin and 2% *v*/*w* pumpkin seed oil). A *t*-test was performed to assess significant differences (*p* < 0.01).

The analysis of the centesimal chemical composition (Table S2) provides fundamental insights into the qualitative characterization of the product, particularly concerning its nutritional aspects. Significant variations were observed among samples from different batches, likely attributable to the presence of inulin and pumpkin seed oil in the formulation. This study highlights the notable impact of these ingredients on both the overall fat content and the fatty acid composition of the samples. Specifically, replacing a portion of pork fat with inulin resulted in a reduction (*p* < 0.01) in total fat content, decreasing from approximately 46% in the control samples to around 31% in the inulin-enriched formulations (Figure 2). This reduction meets the established criteria for the “reduced fat content” nutritional claim, as defined by European food regulations [66].

**Figure 3:**
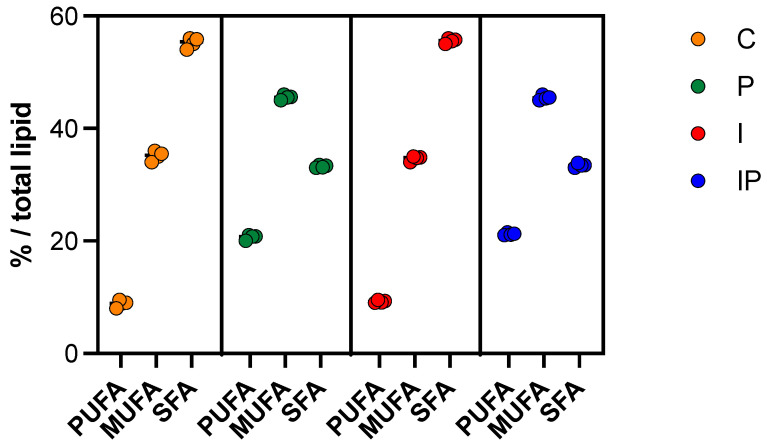
SFA, MUFA, and PUFA content (%/total lipids) in different cooked sausages produced without pumpkin seed oil (C: 10% pork lard; I: 4% pork lard plus 6% *w*/*w* inulin) or with pumpkin seed oil (P: 8% pork lard plus 2% *v*/*w* pumpkin seed oil; IP: 2% pork lard plus 6% *w*/*w* inulin and 2% *v*/*w* pumpkin seed oil). *T*-test was performed to assess significant differences (*p* < 0.01).

From a nutritional perspective, reducing saturated fat intake is widely considered beneficial for cardiovascular health, as excessive consumption strongly correlates with an increased risk of metabolic disorders [67]. In this study, the inclusion of pumpkin seed oil significantly influenced the fatty acid composition of the samples, primarily due to its high content of unsaturated fatty acids, particularly linoleic acid (C18:2) and oleic acid (C18:1). As shown in Figure 3, the use of pumpkin seed oil (batches P and IP) allowed for higher (*p* < 0.01) levels of PUFAs and MUFAs in comparison to those detected in samples from the control batch (C) or in samples obtained using exclusively inulin as a partial fat replacer (I).

**Figure 4:**
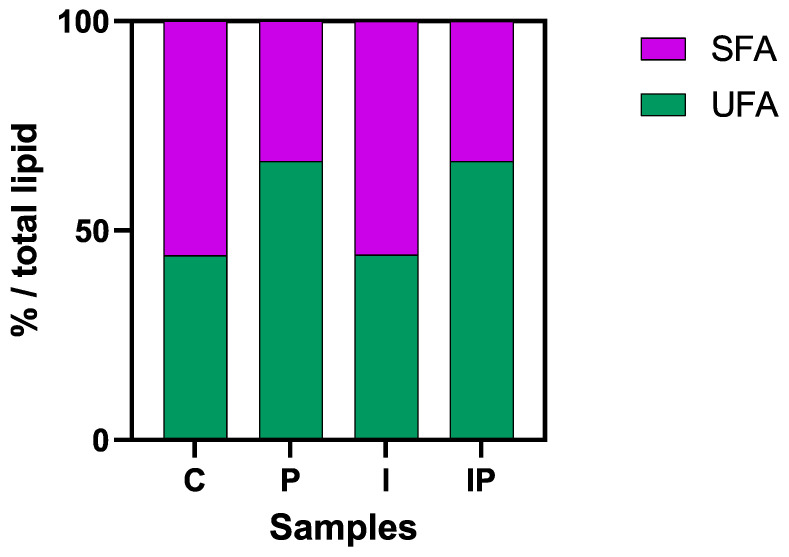
Total lipids reported as percentages of saturated (SFAs) and unsaturated fatty acids (UFAs) (%/total lipids) in different mortadella-style cooked sausages: C (10% pork lard), P (8% pork lard + 2% *v*/*w* pumpkin seed oil), I (4% pork lard + 6% *w*/*w* inulin), and IP (2% pork lard + 6% *w*/*w* inulin + 2% *v*/*w* pumpkin seed oil). SFAs include palmitic (C16:0), stearic (C18:0), and myristic acid (C14:0); UFAs include oleic (C18:1n9c), palmitoleic (C16:1n7), linoleic (C18:2n6c), alpha-linolenic (C18:3n3), and arachidonic acid (C20:4n6).

This compositional shift was anticipated, aligning with previous findings on the lipid-modulating properties of plant-derived oils [68,69,70,71]. Notably, the higher proportion of unsaturated fatty acids in the pumpkin seed oil-enriched samples (Figure 4) enables compliance with the “high unsaturated fat content” claim under EU regulations, which mandates that at least 45% of the total fat content must originate from unsaturated sources.

This modification is nutritionally advantageous, as diets rich in unsaturated fatty acids are associated with anti-inflammatory effects and improved lipid metabolism [67]. Beyond fat reduction, the incorporation of inulin contributed to a substantial increase (*p* < 0.01) in dietary fiber content (Figure 2). In inulin-fortified samples, fiber levels exceeded 15% of the dry matter, meeting the “high fiber content” claim as defined by Regulation (EC) No 1924/2006 [66], which requires a minimum of 6 g of fiber per 100 g of product. This enhancement is particularly relevant from a dietary standpoint, given the well-established benefits of fiber intake, including improved gut health, enhanced satiety, and metabolic regulation [72]. Furthermore, it should be considered that the presence of inulin, a well-documented prebiotic, may also contribute to beneficial changes in the gut microbiota, further enhancing the functional properties of the reformulated product [73,74]. Overall, these findings demonstrate that the combined inulin and pumpkin seed oil facilitates a significant reduction in total fat content, improves the lipid profile, and enhances dietary fiber levels. These compositional changes align with multiple EU nutritional claims and improve the product’s marketability as a healthier alternative to conventional formulations. While previous studies have reported similar benefits of incorporating vegetable oils into meat-based products, concerns regarding oxidative stability and potential impacts on sensory attributes, particularly the development of rancidity, should be carefully considered to ensure long-term consumer acceptance.

### 3.4. Effect on Lipid Oxidation Susceptibility

**Figure 5:**
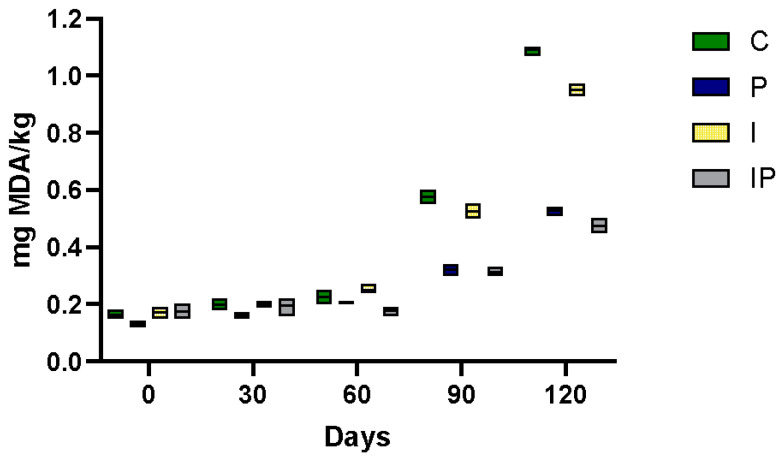
T-BARS values (malondialdehyde mg/Kg) in different cooked sausage samples: C, 10% pork lard; P, 8% pork lard plus 2% *v*/*w* pumpkin seed oil; I, 4% pork lard plus 6% *w*/*w* inulin; IP, 2% pork lard plus6% *w*/*w* inulin and 2% *v*/*w* pumpkin seed oil. The ANOVA test was performed to assess significant differences (*p* < 0.01).

When evaluating and validating technologies for improving the lipid profile, it is crucial to consider their impact on product stability. Adjusting the fatty acid profile, characterized by a higher proportion of unsaturated fatty acids, is nutritionally advantageous because it is associated with better cardiovascular health. However, fatty acid modifications may also increase the product’s susceptibility to lipid oxidation, as unsaturated fatty acids are inherently more prone to oxidative degradation. Consequently, maintaining the oxidative stability of the reformulated product is essential to preserve its quality, sensory attributes, and shelf life [21,75,76]. This process can negatively impact both shelf-life and sensory properties [11,77,78]. Therefore, it is crucial to investigate the effects of pumpkin seed oil incorporation on the oxidative stability of formulations and assess the potential role of its natural antioxidants in mitigating lipid degradation, thereby preserving both nutritional quality and sensory integrity. In our study, as reported in Figure 5, the initial thiobarbituric acid reactive substance (TBARS) levels in cooked buffalo meat products were approximately 0.15 mg malondialdehyde (MDA)/kg at the preparation time, consistent across different batches. Over time, these levels increased significantly, with increments ranging from 0.15 to 0.25 mg MDA/kg after two months of storage. After four months, control samples and those prepared with inulin as a partial substitute for pork fat reached TBARS levels between 0.9 and 1.1 mg MDA/kg. In contrast, samples incorporating pumpkin seed oil consistently exhibited lower TBARS values not exceeding 0.6 mg MDA/kg throughout the storage period.

These findings suggest that pumpkin seed oil may enhance the oxidative stability of buffalo meat products. Previous studies have demonstrated that oils extracted from pumpkin seeds exhibit high resistance to lipid oxidation, attributed to their inherent antioxidant compounds, including phytosterols, such as β-sitosterol, carotenoids, like lutein and zeaxanthin, and phenolic compounds [79,80]. Such studies align with our observations, where the inclusion of pumpkin seed oil resulted in lower TBARS values during storage. The substitution of animal fats with vegetable oils, such as pumpkin seed oil, has been explored in various meat products. For instance, incorporating emulsified melon and pumpkin seed oils in deer burgers improved oxidative stability compared to traditional formulations [41]. Similarly, replacing beef fat with pre-emulsified pumpkin seed oil in chicken meat emulsions enhanced emulsion and oxidative stability [81]. For instance, buffalo meat blocks processed in retort pouches showed TBARS values of 0.24 mg MDA/kg on day 0, increasing to 0.67 mg MDA/kg after 90 days. Research suggests that TBARS values below 1.0 mg MDA/kg typically indicate minimal oxidative degradation, ensuring the meat retains its desired sensory characteristics and taste without significant spoilage or rancidity [82]. Another study observed that minced buffalo meat under high-oxygen modified atmosphere packaging exceeded 1 mg MDA/kg within four days. In contrast, vacuum-packaged samples maintained lower TBARS levels throughout storage [83]. These variations underscore the influence of packaging methods on lipid oxidation. The oxidative stability of meat products is a critical quality attribute that influences shelf life and sensory properties. Lipid oxidation not only deteriorates flavor and color but also poses potential health risks due to the formation of harmful compounds [84]. Therefore, strategies to mitigate lipid oxidation, such as the incorporation of natural antioxidants found in pumpkin seed oil, are of significant interest. Our study demonstrates that incorporating pumpkin seed oil into cooked buffalo meat products effectively reduces lipid oxidation during storage, as evidenced by lower TBARS values. This suggests that pumpkin seed oil is a viable alternative to traditional animal fats, offering nutritional benefits and enhanced oxidative stability. Further research should explore the sensory attributes and consumer acceptance of such formulations to assess their commercial potential fully.

### 3.5. Effect on Sensorial Features

**Figure 6:**
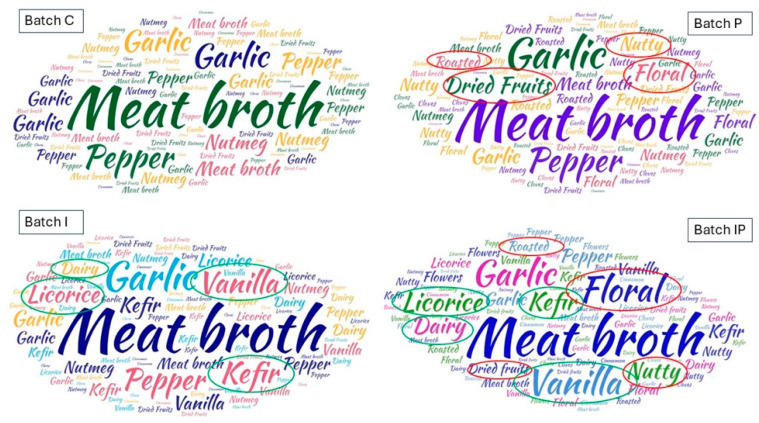
Aroma descriptors’ occurrence in different cooked sausages: C, 10% pork lard; P, 8% pork lard plus 2% *v*/*w* pumpkin seed oil; I, 4% pork lard plus 6% *w*/*w* inulin; IP, 2% pork lard plus 6% *w*/*w* inulin and 2% *v*/*w* pumpkin seed oil.

The sensory evaluation of cooked buffalo meat sausages, conducted using a 9-point scale, revealed significant differences in several sensorial attributes. The replacement of pork fat with inulin (Batch I), pumpkin seed oil (Batch P), or both (Batch IP) influenced the overall sensory profile, highlighting improvements in specific attributes while also introducing some modifications compared to the conventional formulation (Batch C). Panelists reported notable variations in aroma descriptor prevalence among the different formulations. Figure 6 depicts a word cloud representation of the key terms utilized as aroma descriptors in the sensory evaluation. The relative size of each term corresponds to its frequency of occurrence, with larger words indicating descriptors that were more consistently reported by the panelists. This visualization provides insight into the predominant aromatic attributes perceived, highlighting those with the greatest sensory relevance and panelist consensus.

**Figure 7:**
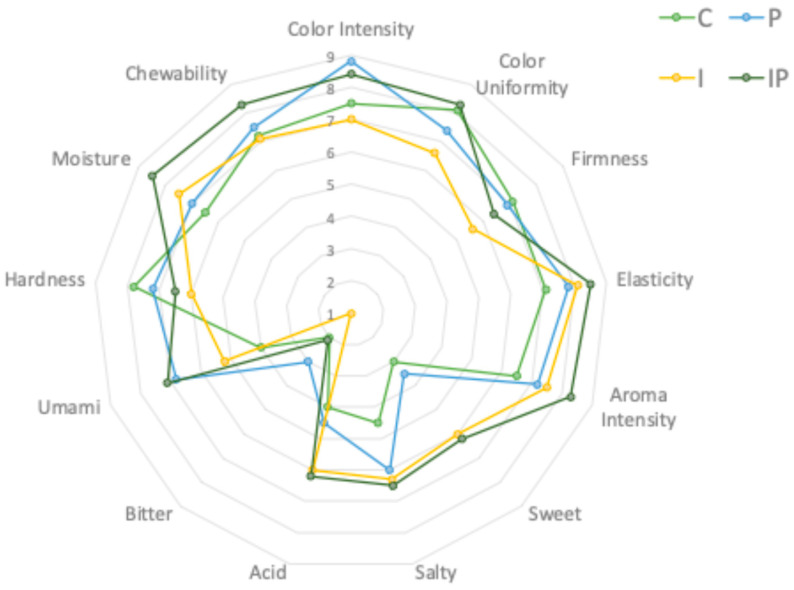
Sensory attributes at the end of storage time (120 days) in different cooked sausage samples: C, 10% pork lard; P, 8% pork lard plus 2% *v*/*w* pumpkin seed oil; I, 4% pork lard plus 6% *w*/*w* inulin; IP, 2% pork lard plus6% *w*/*w* inulin and 2% *v*/*w* pumpkin seed oil.

The control sample (C) exhibited sensory attributes typical of cooked meat products, characterized by meat broth, garlic, and pepper notes. The addition of pumpkin seed oil (P, IP) introduced subtle nutty, roasted, and dried fruit aromas, which panelists described as pleasant and contributing to a more complex sensory profile. This suggests that the oil’s volatile compounds play a role in enhancing the aromatic depth of the product. Furthermore, the incorporation of pumpkin seed oil (P, IP) enriched the overall aromatic complexity by imparting delicate floral nuances. These sensory modifications can be attributed to the oil’s distinctive volatile composition, which includes aldehydes and terpenes, compounds known to contribute to characteristic green and slightly sweet notes in food matrices. This observation is consistent with prior research demonstrating the ability of pumpkin seed oil to introduce mild nutty and toasted aromas in processed meat formulations [85]. Inulin-containing batches (I and IP) showed a slight reduction in the perception of fatty and aged meat aromas, likely due to the lower fat content. Instead, a subtle increase in acidic and dairy-like notes was observed, possibly attributed to inulin’s interaction with moisture retention and flavor compounds [86]. In addition, the inulin-containing samples (I, IP) exhibited additional licorice, kefir, and vanilla-like notes, which were more pronounced in the IP batch, where inulin and pumpkin seed oil acted synergistically. This result is consistent with previous research showing that inulin can influence the sensory perception of fermented and cooked meat products by promoting the release of aromatic compounds typically associated with dairy-like and slightly sweet descriptors [86,87]. Finally, while oxidation-related descriptors (rancid, oxidized) were more pronounced in the control and I sample over storage, they remained lower in P and IP, suggesting a protective effect of pumpkin seed oil, likely due to its antioxidant properties [85]. Flavor, saltiness, acidity, and bitterness were assessed across all samples (Figure 7).

The control (C) had the most balanced flavor profile, with moderate saltiness and mild acidity. The addition of inulin (I and IP) enhanced (*p* < 0.01) the perception of sweetness, in agreement with prior research demonstrating that inulin can contribute to a mild sweet aftertaste in reduced-fat meat products [88]. Pumpkin seed oil-containing samples (P and IP) exhibited an enriched (*p* < 0.01) umami profile with subtle nutty undertones, which some panelists described as “pleasantly complex.” The reduced-fat formulation (I) exhibited a slightly more pronounced acidic perception, likely due to the inulin, which is responsible for water retention, and to the lower fat content, the presence of which is able to mask or balance certain tastes, including acidity. This effect has also been observed in other products [89]. For the color, panelists noted significant differences in intensity and uniformity. While all batches had an appealing color, the incorporation of pumpkin seed oil (P and IP) led to a slight darkening effect, which may be attributed to the natural pigments present in the oil. The inulin-containing samples (I and IP) displayed slightly lighter tones compared to C, which is consistent with findings in other meat products where inulin addition modifies light scattering properties due to changes in fat distribution [90]. In texture attributes, firmness, elasticity, hardness, moisture, and chewability were key parameters in texture evaluation. The control batch (C) exhibited the expected firmness and elasticity typical of conventionally formulated sausages. The partial replacement of pork fat with inulin (I) resulted in a softer texture with increased moisture perception, likely due to inulin’s high water-binding capacity. Samples containing pumpkin seed oil (P and IP) retained a firm structure like C, indicating that the oil contributed to a stable texture, possibly due to its interaction with the protein matrix. However, IP samples exhibited the highest moisture retention and slight chewiness, likely a combined effect of both inulin and oil incorporation. Considering overall sensory acceptability, the IP batch (inulin + pumpkin seed oil) achieved the highest overall acceptability score among the reformulated products. Panelists highlighted its well-balanced sensory attributes, with improved juiciness, mild sweetness, enhanced umami perception, and a complex aroma profile that retained traditional meaty characteristics while introducing mild nutty notes. This suggests that the synergistic effect of inulin and pumpkin seed oil effectively compensates for fat reduction, enhancing sensory properties while potentially improving the product’s nutritional profile. The findings indicate that replacing pork fat with inulin and/or pumpkin seed oil can significantly alter the sensory profile of cooked buffalo meat sausages. While inulin enhances moisture retention and contributes to a slightly sweeter perception, pumpkin seed oil improves oxidative stability and adds mild nutty undertones. The IP formulation (combining inulin and pumpkin seed oil) achieved the best overall sensory balance, suggesting its potential as an optimal strategy for developing healthier meat products without compromising sensory quality. Future research should explore consumer acceptability in broader demographics and investigate the long-term stability of these formulations during storage.

### 3.6. Effect on Microbial Features

**Figure 8:**
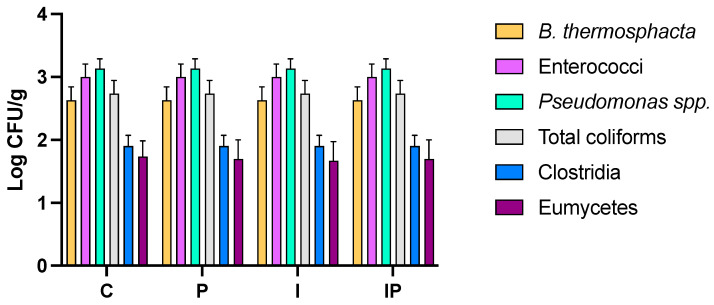
Levels of microbial group in meat mixtures before cooking from batch C, 10% pork lard; P, 8% pork lard plus 2% *v*/*w* pumpkin seed oil; I, 4% pork lard plus 6% *w*/*w* inulin; IP, 2% pork lard plus6% *w*/*w* inulin and 2% *v*/*w* pumpkin seed oil.

The microbiological features of the cooked buffalo meat sausages produced are reported in Figure 8. The results evidenced that the meat mixtures of each batch, prior to cooking, are contaminated with several microbial groups, including gram-positive (*B. thermoshacta*, Enterococci, and *Clostridia*), gram-negative (*Pseudomonas* and total coliforms), as well as eumicetes.

The combination of thermal treatment, rapid chilling, and subsequent pasteurization effectively reduced microbial loads to undetectable levels in all samples immediately after processing (Table S3). Throughout the first 90 days of storage, no viable microorganisms were detected in any batch, confirming the efficacy of these processing steps in microbial control. At 120 days, *Clostridium* spp. was detected at low levels exclusively in the conventional (C) and inulin-containing (I) samples, whereas no detectable *Clostridia* were found in the samples containing pumpkin seed oil (P and IP). These findings are consistent with studies reporting that cooking followed by rapid chilling significantly reduces bacterial populations, including spoilage and pathogenic microorganisms, by denaturing proteins and disrupting cellular structures. However, the late-stage detection of *Clostridium* spp. in the C and I batch suggests that spores may have survived the thermal process and germinated under favorable anaerobic conditions over time. The absence of *Clostridium* spp. in the P and IP samples indicates that pumpkin seed oil may exert an inhibitory effect on spore germination and growth. This aligns with previous research demonstrating the antimicrobial properties of pumpkin seed oil, attributed to bioactive compounds such as tocopherols, carotenoids, and polyphenols, which have been shown to exert bacteriostatic and bactericidal effects against *Clostridia* and other anaerobic bacteria [91,92]. The presence of *Clostridium* spp. in the inulin-containing samples but not in those with pumpkin seed oil highlights the distinct influence of these ingredients on microbial stability. Inulin, while widely recognized as a fermentable prebiotic fiber for specific microbial groups, such as lactic acid bacteria and Bifidobacteria, does not exert a direct inhibitory effect on *Clostridium* spp. In our study, its inclusion primarily contributed to increased moisture retention and texture modification, which may have inadvertently created more favorable conditions for the growth of anaerobic spore-forming bacteria. This observation underscores that, despite its functional and technological benefits, inulin alone is insufficient to suppress *Clostridium* proliferation in processed meat products. Therefore, it reinforces the need to combine inulin with bioactive antimicrobial compounds when designing reformulated meats aimed at improved microbial safety and extended shelf life. The antimicrobial activity of pumpkin seed oil presents a promising approach to improving the microbial stability of cooked meat products, supporting its potential application as a natural preservative in reformulated meat products. Further studies should explore the mechanisms underlying this inhibition and assess its effectiveness under different storage conditions to optimize shelf-life extension in minimally processed meat products.

## 4. Conclusions

The reformulation of mortadella-style cooked sausages with inulin and pumpkin seed oil led to significant improvements in nutritional profile, oxidative stability, sensory quality, and microbial safety. The reduction in pork fat increased the fiber content and improved the fatty acid profile, while pumpkin seed oil enhanced antioxidant protection, keeping lipid peroxidation low throughout storage. Sensory analysis revealed enriched aromatic complexity—floral, herbal, and lactic notes—without compromising acceptability. Microbiological results confirmed process efficacy, with Clostridium spp. undetectable in samples containing pumpkin seed oil after 120 days, suggesting potential antimicrobial effects. These findings underscore the value of plant-derived ingredients as natural tools to improve product quality and shelf life. In a context of rising antimicrobial resistance, this strategy offers the meat industry a sustainable and consumer-aligned alternative to synthetic additives, with strong potential for technological transfer and innovation.

## Data Availability

The original contributions presented in this study are included in the article/Supplementary Materials. Further inquiries can be directed to the corresponding authors.

## Supplementary Materials

The following supporting information can be downloaded at https://www.mdpi.com/article/10.3390/foods14081427/s1, Table S1. Sensory analysis form; Document S1. Consent Sensory Evaluation; Table S2. Chemical composition in different cooked sausage samples: C, 10% pork lard; P, 8% pork lard plus 2% *v*/*w* pumpkin seed oil; I, 4% pork lard plus 6% *w*/*w* inulin; IP, 2% pork lard plus 6% *w*/*w* inulin and 2% *v*/*w* pumpkin seed oil. The ANOVA test was performed to assess significant differences (*p* < 0.01). C control cooked sausages: P, supplemented with 2% pumpkin seed oil; I, supplemented with 6% inulin; and IP, supplemented with 2% pumpkin oil and 6% inulin. Table S3. Microbial levels (log UFC/g) in different cooked sausage samples: C, 10% pork lard; P, 8% pork lard plus 2% *v*/*w* pumpkin seed oil; I, 4% pork lard plus 6% *w*/*w* inulin; IP, 2% pork lard plus6% *w*/*w* inulin and 2% *v*/*w* pumpkin seed oil.
