# Supplementary material for: Use of Inulin and Pumpkin Oil in the Manufacture of High-Quality Mortadella-Style Sausage from Buffalo Meat
Source: Foods. 2025 Apr 21;14(8):1427. doi: 10.3390/foods14081427 (PMC12027086; doi:10.3390/foods14081427)
Supplement: Supplementary file 1 [file foods-14-01427-s001.zip › foods-3572098-supplementary.pdf]

Document S1. Consent Sensory Evaluations

Dear Sensory Panelist,

People 18 years of age and older are being invited to participate in a research study to evaluate Mortadella-Style Sausage from Buffalo Meat. This project is being conducted by Prof. Patrizio Tremonte from Department of Agricultural, Environmental and Food Sciences at University of Molise

PURPOSE OF THE STUDY: The sensory analyses are conducted by trained volunteers and are part of a larger study to investigated the effects of replacing pork fat with inulin and pumpkin seed oil in a cooked buffalo meat product.

DETAILS:

- Dates: Each volunteer will participate in 10 training sessions, ensuring consistency and reliability in their assessments. Each judge was anonymously presented with one sample per batch, consisting of a single slice of cooked sausage and conducts a threefold sensory analysis on the bar using a 9-point scale.
- Time Commitment: Each volunteer panelist participates in 10 training sessions and 3 sensory analysis sessions. Each session duration is 60 minutes.
- Information Location: Sensory analyses are conducted at the Department's facilities

EXPLANATION OF PROCEDURES: Panelist conducts a threefold sensory analysis on the bar using a 9-point scale to assess appearance, color, aroma, flavor, texture, stickiness, aftertaste and overall liking.
VOLUNTARITY: participation is voluntary, and the panelist may withdraw at any time without penalty.
Your help will be greatly appreciated in making this study meaningful.
PRIVACY AND CONFIDENTIALITY: All information obtained during the testing procedures will remain confidential and will not be visible to other panelists or persons not associated with this study. The identity of the panelist will not be revealed in the results of the experiment. Only comparisons will be made and reported in summary form. The data will only be accessible to the researchers of the study and may only be used for the purpose of scientific publications in journals intended for the scientific community.
POTENTIAL BENEFITS AND RISKS: Participation in the study does not result in any direct benefits but does produce useful new knowledge in the field of food security.
CONTACT INFORMATION: If you have any questions about this project, please contact me via email tremonte@unimol.it or dr. Silvia Jane Lombardi silvia.lombardi@unimol.it.

You will be given a copy of this consent form to keep.

______________________________________   __________________

Your Signature   Date

Table S1: Sensory analysis form

**Judge:**	**Sample:**								
**Aroma**									
Intensity	1	2	3	4	5	6	7	8	9
	Meat family								
	Fresh meat			Meat broth			Fat		
	Aged meat				Other____________________				
	Animal Family								
	Stable		Gut						
	Leather		Other ________________						
Dominant Descriptor	Spicy Family								
	Green pepper		Garlic		Cinnamon			Red pepper	
	Nutmeg				Other____________________				
	Other								
	Dried fruit		Dairy ---Kefir			Mold			
	Rancid		Floral Vanilla --- Licorice			Other ________			
**Color**									
Intensity	1	2	3	4	5	6	7	8	9
	Pale Pink	Intense pink							
Uniformity	1	2	3	4	5	6	7	8	9
	Not uniform								Uniform
**STRUCTURE**	**Visual perception**								
Compactness	1	2	3	4	5	6	7	8	9
Elasticity	1	2	3	4	5	6	7	8	9
Crushing	1	2	3	4	5	6	7	8	9
**Aroma**									
Intensity	1	2	3	4	5	6	7	8	9
	Meat family								
	Fresh meat			Sour meat			Fat		
	Aged meat				Other____________________				
	Animal Family								
	Stable		Casing						
	Leather		Other ______________________________						
Dominant Descriptor	Spicy Family								
	Pepper		Garlic		Cinnamon			Bell pepper	
	Nutmeg			Other_____________________________					
	Off-Flavor								
	Acetic		Oxidized			Mold			
	Rancid		Ammonia			Other __________________			
**Taste**									
Sweet	1	2	3	4	5	6	7	8	9
Salty	1	2	3	4	5	6	7	8	9
Acidic	1	2	3	4	5	6	7	8	9
Bitter	1	2	3	4	5	6	7	8	9
Umami	1	2	3	4	5	6	7	8	9

**TRIGEMINAL SENSATION**									
Spicy	1	2	3	4	5	6	7	8	9
**STRUCTURAL ATTRIBUTES**									
Hardness	1	2	3	4	5	6	7	8	9
Moisture	1	2	3	4	5	6	7	8	9
Chewability	1	2	3	4	5	6	7	8	9

Table S2. Chemical composition in different cooked sausage samples: C, 10% pork lard; P, 8% pork lard plus 2% v/w pumpkin seed oil; I, 4% pork lard plus 6% w/w inulin; IP, 2% pork lard plus 6% w/w inulin and 2% v/w pumpkin seed oil. Anova test was performed to assess significant differences (p < 0.01). C control cooked sausages; P, supplemented with 2% pumpkin seed oil; I, supplemented whit 6% inulin; and IP, supplemented with 2% pumpkin oil and 6% inulin

	C	P	I	IP
Moisture (%)	76^{a}	76^{a}	75.34^{a}	75.34^{a}
Protein (%/d.m.)	42.71^{a}	42.71^{a}	41.57^{a}	41.57^{a}
NPN (%/d.m.)	2.08^{a}	2.08^{a}	2.03^{a}	2.03^{a}
Ash (%/d.m.)	7.92^{a}	7.92^{a}	7.06^{a}	7.062^{a}
Fibre (%/d.m.)	0.00	0.00	15.90^{a}	15.90^{a}
Carbohydrates (%/d.m.)	1.25^{a}	1.25^{a}	1.22^{b}	1.22^{b}
Lipid (%/d.m.)	46.04^{a}	46.04^{a}	32.20^{a}	32.20^{a}
SFA (% d.m.)	24.57^{a}	14.40^{b}	17.96^{a}	11.01^{b}
MUFA (% d.m.)	17.44^{a}	21.63^{a}	11.17^{a}	13.28^{a}
PUFA (% d.m.)	4.03^{a}	10.02^{b}	3.06^{a}	7.90^{c}
PUFA/SFA (% d.m.)	0.16^{a}	0.69^{b}	0.17^{a}	0.71^{b}

According to the ANOVA statistical test, values within a row with different letters are significantly different ($p < 0.01$).

Table S3. Microbial levels (log UFC/g) in different cooked sausage samples: C, 10% pork lard; P, 8% pork lard plus 2% v/w pumpkin seed oil; I, 4% pork lard plus 6% w/w inulin; IP, 2% pork lard plus6% w/w inulin and 2% v/w pumpkin seed oil.

**Microorganisms**	**C**						**P**						**I**						**IP**					
	**before cooking**	**0**	**30**	**60**	**90**	**120**	**before cooking**	**0**	**30**	**60**	**90**	**120**	**before cooking**	**0**	**30**	**60**	**90**	**120**	**before cooking**	**0**	**30**	**60**	**90**	**120**
B. thermospacta	2.62 ± 0.19	ND	ND	ND	ND	ND	2.58 ± 0.18	ND	ND	ND	ND	ND	2.63 ± 0.15	ND	ND	ND	ND	ND	2.72 ± 0.14	ND	ND	ND	ND	ND
**Enterococci**	3.02 ± 0.16	ND	ND	ND	ND	ND	3.04 ± 0.12	ND	ND	ND	ND	ND	2.99 ± 0.18	ND	ND	ND	ND	ND	3.23 ± 0.15	ND	ND	ND	ND	ND
Pseudomonas spp	3.12 ± 0.19	ND	ND	ND	ND	ND	3.14 ± 0.20	ND	ND	ND	ND	ND	3.08 ± 0.21	ND	ND	ND	ND	ND	3.14 ± 0.21	ND	ND	ND	ND	ND
**Total coliforms**	2.7 ± 0.15	ND	ND	ND	ND	ND	2.73 ± 0.18	ND	ND	ND	ND	ND	2.37 ± 0.18	ND	ND	ND	ND	ND	2.69± 0.17	ND	ND	ND	ND	ND
Clostridia	1.90 ± 0.14	ND	ND	ND	ND	1.40 ± 0.23	1.85 ± 0.24	ND	ND	ND	ND	ND	1.42 ± 0.16	ND	ND	ND	ND	1.53 ± 0.21	1.92 ± 0.13	ND	ND	ND	ND	1.90 ± 0.14
Eumycetes	1.70 ± 0.11	ND	ND	ND	ND	ND	1.74 ± 0.11	ND	ND	ND	ND	ND	1.56 ± 0.14	ND	ND	ND	ND	ND	1.74 ± 0.09	ND	ND	ND	ND	ND

ND: non-detectable count level
